# Real-world experience with efgartigimod in generalized myasthenia gravis: a single-center retrospective study in China

**DOI:** 10.3389/fimmu.2026.1752846

**Published:** 2026-04-27

**Authors:** Zhenyu Niu, Jianchun Wang, Jingru Ren, Ran Liu, Jing Guo, Nan Zhang, Yiming Zheng, Hongjun Hao, Feng Gao, Haiqiang Jin

**Affiliations:** Department of Neurology, Peking University First Hospital, Beijing, China

**Keywords:** efgartigimod, generalized myasthenia gravis, individualized treatment, real-world study, reduced-frequency regimen

## Abstract

**Objective:**

This study aimed to evaluate the real-world efficacy, safety, and application patterns of efgartigimod in Chinese patients with generalized myasthenia gravis (gMG), with a focus on individualized treatment strategies.

**Methods:**

We conducted a single-center, retrospective analysis of 81 gMG patients who received at least one standard cycle of efgartigimod (10 mg/kg weekly for 4 weeks). Disease severity was assessed using Myasthenia Gravis Activities of Daily Living (MG-ADL), Quantitative Myasthenia Gravis (QMG), and Myasthenia Gravis Composite (MGC) scores at baseline, week 2, and week 4. Retreatment and regimen modifications were individualized, which included exploration of a reduced-frequency regimen (2 infusions every 2 weeks) in selected patients.

**Results:**

In the overall cohort, 65 (80.25%) and 63 (77.78%) patients achieved MG-ADL (reduction ≥2) and QMG (reduction ≥3) responses, respectively, after the first cycle. 65 (80.25%) patients received only one cycle; among them, 41 (63.07%) maintained disease control with concomitant immunosuppressive therapies without requiring additional efgartigimod. The 16 patients receiving multiple cycles (≥2) had higher baseline disease severity (higher Myasthenia Gravis Foundation of America class, MG-ADL, and QMG scores) and showed progressive improvement with flexible retreatment. In a selected subgroup of 9 patients (based on age, robust response, and significant IgG reduction), a reduced-frequency regimen (2 infusions every 2 weeks) maintained efficacy while reducing the infusion burden. Adverse events were mostly mild, with no new safety signals.

**Interpretation:**

Efgartigimod demonstrated rapid and broad efficacy in a real-world gMG cohort. An “induction-maintenance” strategy, using efgartigimod for acute control and conventional immunosuppressants for long-term management, proved effective in most patients. A reduced-frequency regimen may be feasible in selected cases, highlighting the value of individualized treatment approaches in optimizing outcomes and reducing treatment burden.

## 1 Introduction

Myasthenia gravis (MG) is an autoimmune disorder affecting the neuromuscular junction, primarily mediated by autoantibodies targeting postsynaptic membrane proteins (1). These include antibodies against the acetylcholine receptor (AChR-Ab, the most common, detected in >85% of patients), muscle-specific tyrosine kinase (MuSK-Ab), or lipoprotein-related protein 4 (LRP4-Ab). A smaller proportion of patients are seronegative for these antibodies (2). Efgartigimod, a human immunoglobulin G1 (IgG1) Fc fragment, has demonstrated efficacy and safety by promoting the degradation of IgG in patients with generalized MG (gMG) across various antibody profiles in multiple clinical trials and cohort studies (3–6), with reported response rates ranging from 64% to 97% (6–9). Consequently, it has been approved and widely adopted in many countries and is recommended in several guidelines for both rapid induction and maintenance therapy in gMG (10, 11).

Despite its clinical benefits, the use of efgartigimod presents new challenges, particularly regarding dosing strategies. The standard regimen consists of weekly infusions for four weeks per treatment cycle, with a minimum one-week interval between cycles (5). However, due to its nonspecific reduction of IgG levels, some patients experience symptom recurrence during the treatment-free intervals as autoantibodies are replenished, preventing them from sustaining a minimal manifestation status and impairing quality of life (6, 8, 12). Although long-term, multi-cycle efgartigimod treatment has been shown to be safe and effective, frequent infusions pose logistical and economic burdens. Thus, key clinical questions have emerged concerning the optimal integration of efgartigimod with conventional first- and second-line therapies—such as corticosteroids, immunosuppressants (ISTs), and rituximab—particularly in newly diagnosed patients, and when efgartigimod treatment may be reasonably concluded.

This study describes a single-center, real-world experience of efgartigimod use in Chinese patients with gMG. In our cohort, most patients did not receive long-term multi-cycle efgartigimod monotherapy. Instead, efgartigimod was often used to manage acute symptoms, while corticosteroids, ISTs, or rituximab were concurrently administered for long-term relapse prevention. Furthermore, in selected patients with stable conditions, we explored modified efgartigimod dosing intervals to sustain clinical benefits while minimizing immunosuppressive impact and infusion frequency. The aim of this study is to contribute to the development of individualized immunotherapy strategies for gMG.

## 2 Methods

### 2.1 Study design and patients included

This single-center, retrospective analysis consecutively enrolled patients with a confirmed diagnosis of gMG admitted to Peking University First Hospital between November 1, 2023 and July 31, 2025 and who completed at least one full standard treatment cycle of efgartigimod (one infusion per week for four weeks). Clinical data were collected before and after each treatment cycle. The data cut−off date for analysis was November 15, 2025, ensuring a minimum follow−up of 3 months for the last enrolled patient.

All patients included in this study met the diagnostic criteria established by the internationally recognized 2022 Japanese Clinical Guidelines for Myasthenia Gravis (10). This required the presence of fluctuating skeletal muscle weakness and fatigability, along with the detection of pathogenic autoantibodies including AChR-Ab, MuSK-Ab and LRP4-Ab and/or supportive evidence of impaired neuromuscular transmission (2). Confirmatory tests included a positive neostigmine test, a decremental response (>10%) in compound muscle action potentials (CMAP) on low-frequency repetitive nerve stimulation (RNS), or increased jitter on single-fiber electromyography (SFEMG). Pathogenic autoantibodies were detected using enzyme-linked immunosorbent assay (ELISA) with widely validated commercial kits. In addition to the three primary pathogenic autoantibodies, testing for acetylcholinesterase antibody (AChE-Ab), Titin antibody (Titin-Ab), and ryanodine receptor antibody (RyR-Ab) was also performed. Total serum IgG levels were measured using immunoturbidimetry on a Beckman Coulter IMMAGE 800 analyzer (Beckman Coulter, Brea, CA, USA).

Disease severity was classified according to the Myasthenia Gravis Foundation of America (MGFA) clinical classification. Baseline disease severity and activity were further quantified using Myasthenia Gravis Activities of Daily Living (MG-ADL) scale, Quantitative Myasthenia Gravis (QMG) score, and Myasthenia Gravis Composite (MGC) score, in accordance with international standards (13, 14). All patients provided signed informed consent forms, and the study protocol was approved by the Institutional Review Board of Peking University First Hospital.

### 2.2 Treatment protocol and clinical assessments

All enrolled patients received the standard efgartigimod regimen of 10 mg/kg administered by intravenous infusion once per week for four consecutive weeks. Disease severity, as measured by the MG-ADL, QMG, and MGC scores, was assessed at baseline (defined as Week 0) and subsequently at one week after the second infusion (Week 2) and one week after the fourth infusion (Week 4). A favorable treatment response was predefined as a reduction of >2 points in the MG-ADL score or a reduction of >3 points in the QMG score from baseline to Week 4, which also included patients achieved the status of Clinical Minimal Manifestations (CMI).

The initiation of subsequent treatment cycles was individualized. Objective criteria informed by the ADAPT+ study and other real-world evidence were considered. These criteria included a time interval of at least 4 weeks since the last cycle, an MG-ADL total score of ≥5 (with >50% of the points derived from non-ocular items), and an improvement of <2 points in the MG-ADL score compared to the baseline of the current cycle. The final decision on whether and when to commence a new cycle was made by the treating physician based on these objective parameters, the patient’s clinical status and evidence of relapse, as well as patient preference, rather than a fixed, pre-scheduled protocol. Patients who received two or more treatment cycles similarly underwent repeated MG-ADL, QMG, and MGC assessments at baseline (pre-cycle), one week after the second infusion (Week 2), and one week after the fourth infusion (Week 4) of each cycle. Furthermore, for some patients with stable clinical conditions and based on personal preference, a modified maintenance regimen was implemented after one or several standard cycles. This regimen consisted of a reduced infusion frequency of once every two weeks for a total of two infusions per cycle (each at the standard 10 mg/kg dose). These patients also underwent the same schedule of repeated clinical assessments (MG-ADL, QMG, MGC) at baseline and one week after each infusion (Week 2 and Week 4 of the cycle). In this study, all outcome assessments were performed by the same dedicated panel of three qualified clinicians who had not taken part in the research in order to ensure consistency.

Concomitant ISTs were maintained at stable doses throughout the efgartigimod treatment cycle unless safety concerns required adjustment. The following dose ranges were used: oral corticosteroids (prednisone 5–40 mg/day or equivalent), mycophenolate mofetil (1000–1500 mg/day), tacrolimus (2–4 mg/day with target trough levels of 4–8 ng/mL), methotrexate (10–15 mg/week), and azathioprine (1–2 mg/kg/day). Rituximab was administered as 375 mg/m^2^ weekly for 4 weeks and 375 mg/m^2^ every 6 months subsequently for maintenance. No dose escalation of ISTs was performed during the efgartigimod cycle to allow independent assessment of efgartigimod’s effect.

### 2.3 Statistical analysis

Given that a substantial proportion of patients did not receive a second or subsequent cycle of efgartigimod after completing the initial one, the dataset inherently contained missing data for long-term efficacy observations. To ensure analytical rigor, the following strategies were employed:

1. The Full Analysis Set (FAS), comprising all patients who received at least one full standard treatment cycle, was used for the analysis of short-term safety, efficacy within the first cycle, and baseline characteristics. The Long-term Efficacy Analysis Set, defined as patients who completed two or more treatment cycles with robust follow-up data, was utilized to describe long-term treatment patterns, inter-cycle intervals, and the durability of the treatment effect. This approach aimed to prevent overinterpretation based on incomplete data.
2. A detailed attribution analysis was conducted on the subgroup of patients who received only one treatment cycle to illustrate patient disposition throughout the study.
3. A sensitivity analysis was performed to compare the Week 4 treatment response (end of first cycle) between patients who received only one cycle and those who proceeded to multiple cycles, thereby assessing potential differences in the initial treatment characteristics between these groups.

All statistical analyses were performed using the R programming language. Figures were generated using either the ggplot2 package in R or GraphPad Prism 9. Normality of continuous variables was assessed using the Shapiro−Wilk test. Results are presented as mean ± SD for normally distributed data, or as median (Q1, Q3) for non−normally distributed data. Normally distributed data were compared using T−test, and non−normally distributed data were compared using the Mann−Whitney U test. Given the limited sample size, Fisher’s exact test was used for comparing categorical variables between groups. P-value < 0.05 was considered statistically significant.

## 3 Results

### 3.1 Demographic and clinical profiles of patients

A total of 81 patients with gMG were included in this analysis. The cohort was subsequently divided into two groups for comparative purposes: those who received only one treatment cycle (n=65) and those who received two or more cycles (n=16). The cohort had a median age of 67 years and was predominantly female (69.14%). No significant differences were observed between the two groups in terms of gender, age, or disease duration. The baseline demographic and clinical characteristics of the total population and these subgroups are summarized in **Table 1**.

**Table 1:** Baseline demographic and clinical characteristics of the study population.

Features	Total (N = 81)	1 cCycle EFG (N = 65)	≥ 2 cycles EFG (N = 16)	*P*-value
Gender				
Female	56 (69.14%)	43 (66.15%)	13 (81.25%)	0.367
Male	25 (30.86%)	22 (33.85%)	3 (18.75%)	
Age [median, (Q1, Q3)]	67 (55, 76)	67 (55, 76)	62 (58, 78)	0.361
Time since diagnosis				
< 1 year	26 (32.10%)	22 (33.85%)	4 (25.00%)	0.846
1–5 years	30 (37.04%)	24 (36.92%)	6 (37.50%)	
5–10 years	13 (16.05%)	10 (15.38%)	3 (18.75%)	
More than 10 years	12 (14.81%)	9 (13.85%)	3 (18.75%)	
MGFA classification				
IIa	31 (38.27%)	27 (41.54%)	4 (25.00%)	0.046*
IIb	24 (29.63%)	19 (29.23%)	5 (31.25%)	
IIIa	4 (4.94%)	3 (4.62%)	1 (6.25%)	
IIIb	17 (20.99%)	14 (21.54%)	3 (18.75%)	
IVa	1 (1.23%)	1 (1.54%)	0 (0.00%)	
IVb	3 (3.70%)	1 (1.54%)	2 (12.50%)	
V	1 (1.23%)	0 (0.00%)	1 (6.25%)	
Pathogenic autoantibody				
AChR-Ab	75 (92.59%)	60 (92.31%)	15 (93.75%)	1.000
MuSK-Ab	4 (4.94%)	3 (4.62%)	1 (6.25%)	
AChR-Ab + MuSK-Ab	1 (1.23%)	1 (1.54%)	0 (0.00%)	
Negative	1 (1.23%)	1 (1.54%)	0 (0.00%)	
Other autoantibody				
AChE-Ab	11 (13.58%)	8 (12.31%)	3 (18.75%)	0.447
Titin-Ab	56 (69.14%)	44 (67.69%)	12 (75.00%)	0.765
RyR-Ab	13 (16.05%)	9 (13.85%)	4 (25.00%)	0.275
Previous thymectomy	17 (20.99%)	16 (24.62%)	1 (6.25%)	0.171
Oral corticosteroids	61 (75.31%)	48 (73.85%)	13 (81.25%)	0.749
ISTs used prior to EFG				
Methotrexate	4 (4.94%)	3 (4.62%)	1 (6.25%)	1.000
Mycophenolate Mofetil	19 (23.46%)	13 (20.00%)	6 (37.50%)	0.187
Tacrolimus	26 (32.10%)	18 (27.69%)	8 (50.00%)	0.133
Azathioprine	1 (1.23%)	1 (1.54%)	0 (0.00%)	1.000
Rituximab	11 (13.58%)	9 (13.85%)	2 (12.50%)	1.000
Myasthenic crisis history	16 (19.75%)	12 (18.46%)	4 (25.00%)	0.726
Tumor (exclude thymoma)	16 (19.75%)	12 (18.46%)	4 (25.00%)	0.726

EFG, efgartigimod; MGFA, Myasthenia Gravis Foundation of America; AChR-Ab, acetylcholine receptor antibody; MuSK-Ab, muscle-specific tyrosine kinase antibody; LRP4-Ab, lipoprotein-related protein 4 antibody; AChE-Ab, acetylcholinesterase antibody; RyR-Ab, ryanodine receptor antibody; ISTs, immunosuppressants. *p < 0.05 was considered statistically significant.

A statistically significant difference was found in the baseline MGFA clinical classification between the groups (p=0.046). The multi-cycle group contained a higher proportion of patients with more severe disease (MGFA class IVb and V) compared to the single-cycle group. Serologically, the vast majority of patients (92.59%) were positive for AChR-Ab, with no significant differences in the distribution of pathogenic autoantibodies between the groups. The prevalence of other autoantibodies, including AChE-Ab, Titin-Ab, and RyR-Ab, was also comparable. The groups were comparable in their treatment history, including prior thymectomy (24.62% vs. 6.25%; p=0.171), myasthenic crisis (18.46% vs. 25.00%; p=0.726), and baseline corticosteroid use (73.85% vs. 81.25%; p=0.749). Prior use of immunosuppressants and rituximab also showed no significant differences (all p > 0.05). The median follow-up duration for the entire cohort was 10.2 months (IQR, 5.8-16.5 months). For patients who received only one cycle (n=65), the median follow-up was 9.5 months (IQR, 5.0-15.8 months).

### 3.2 Treatment response during the first cycle

At baseline, the total cohort (N = 81) presented with mean scores of 8.08 ± 4.28 on the MG-ADL, 13.50 ± 8.05 on the MGC, and 14.80 ± 5.57 on the QMG. Patients who subsequently received multiple treatment cycles (≥2 Cycles EFG) exhibited significantly higher baseline disease severity, as reflected by MG-ADL (9.87 ± 4.79 vs. 7.64 ± 4.06, p=0.046) and QMG scores (17.37 ± 6.12 vs. 14.16 ± 5.29, p=0.038), compared to those receiving only one cycle.

**Figure 1:**
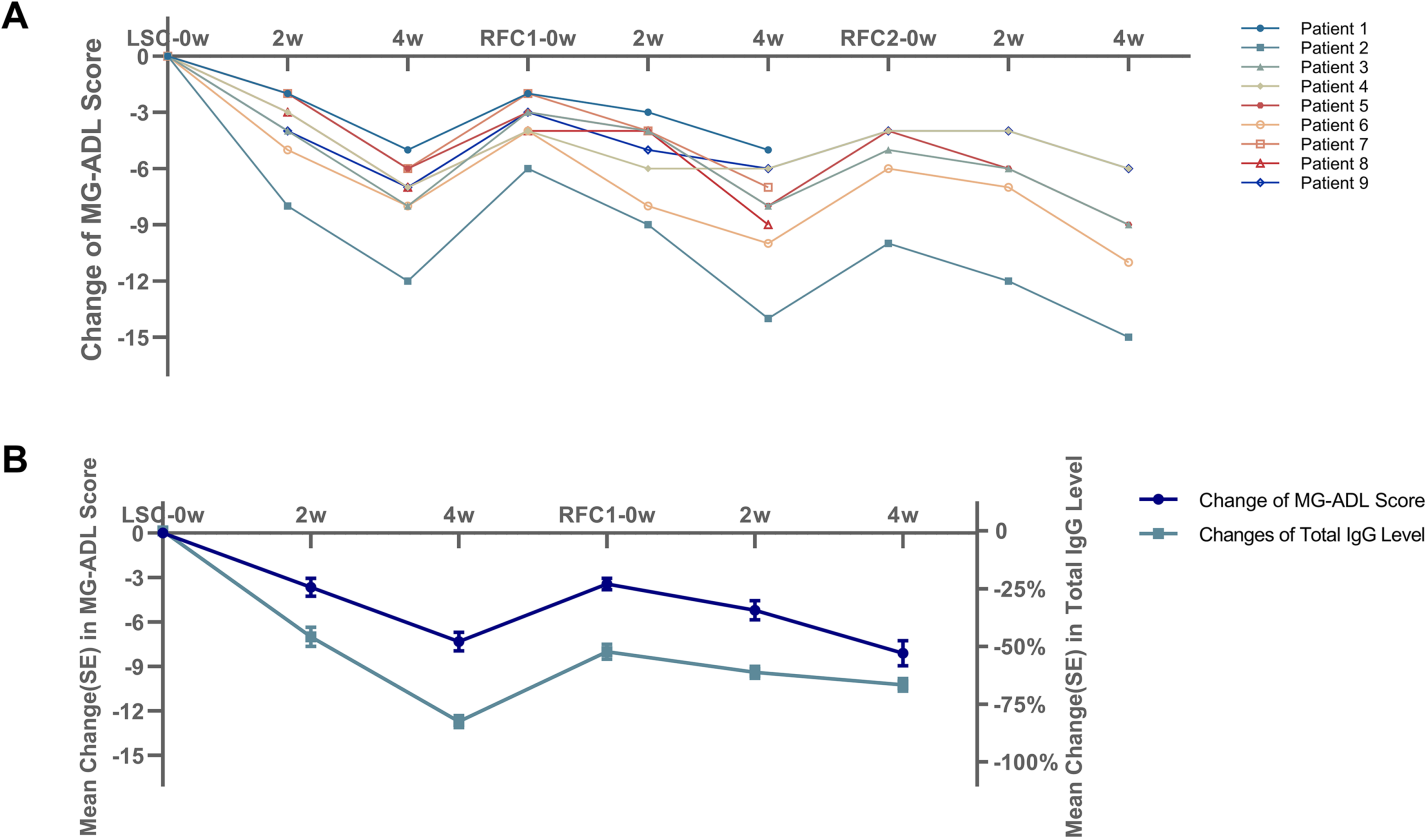
Clinical outcomes and IgG levels of the reduced-frequency efgartigimod regimen. **(A)** MG-ADL score dynamics for the 9 patients who transitioned from a standard cycle to the reduced-frequency regimen (2 infusions administered every two weeks). The figure illustrates the baseline score before the last standard cycle (LSC), the score after the LSC, the baseline before the first reduced-frequency cycle (RFC 1), the score after RFC 1, and (for the 6 patients who received it) the score after the second reduced-frequency cycle (RFC 2). **(B)** Changes in total IgG levels (expressed as percentage change from LSC−0W baseline) and MG−ADL scores in patients receiving the reduced−frequency regimen. Notes: MG-ADL, Myasthenia Gravis Activities of Daily Living; LSC, Last Standard Cycle; RFC, Reduced-Frequency Cycle.

Significant clinical improvements were observed across the entire cohort following efgartigimod treatment. The overall temporal evolution of score reductions is visually represented in **Figure 1A**. At Week 2, mean score reductions were -2.06 ± 1.80 (MG-ADL), -2.97 ± 3.68 (MGC), and -2.72 ± 2.51 (QMG). By Week 4, improvements were more pronounced, with mean reductions of -3.76 ± 2.98 (MG-ADL), -6.46 ± 6.13 (MGC), and -5.33 ± 4.45 (QMG). The response rates at Week 4 were 80.25% (65/81) based on the MG-ADL criterion (reduction ≥2) and 77.78% (63/81) based on the QMG criterion (reduction ≥3). Patients in the multi-cycle group demonstrated numerically greater mean score reductions at both Week 2 and Week 4 compared to the single-cycle group, with the differences in MGC and QMG score changes reaching statistical significance at Week 4 (p<0.05). All 16 patients reached at least one score-defined CMI. Data are summarized in **Table 2**.

**Table 2:** Treatment response following the first cycle of efgartigimod.

	Total (N = 81)	1 Cycle EFG (N = 65)	≥ 2 Cycles EFG (N = 16)	*P*-value
Baseline scores (Mean ± SD)				
MG-ADL	8.08 ± 4.28	7.64 ± 4.06	9.87 ± 4.79	0.046*
MGC	13.50 ± 8.05	12.67 ± 7.43	16.87 ± 9.76	0.061
QMG	14.80 ± 5.57	14.16 ± 5.29	17.37 ± 6.12	0.038*
2W Score changes (Mean ± SD)				
MG-ADL	-2.06 ± 1.80	-1.93 ± 1.58	-2.56 ± 2.47	0.216
MGC	-2.97 ± 3.68	-2.43 ± 2.83	-5.18 ± 5.62	0.007**
QMG	-2.72 ± 2.51	-2.55 ± 2.39	-3.44 ± 2.92	0.210
2W Response rate				
MG-ADL (reduce ≥ 2)	51 (62.96%)	41 (63.08%)	10 (62.50%)	0.360
QMG (reduce ≥ 3)	41 (50.61%)	30 (46.15%)	10 (62.50%)	0.276
4W Score Changes (Mean ± SD)				
MG-ADL	-3.76 ± 2.98	-3.33 ± 2.56	-5.50 ± 3.89	0.009**
MGC	-6.46 ± 6.13	-5.47 ± 5.11	-10.50 ± 8.20	0.003**
QMG	-5.33 ± 4.45	-4.72 ± 4.37	-7.81 ± 4.02	0.012*
4W Response rate				
MG-ADL (reduce ≥ 2)	65 (80.25%)	50 (76.92%)	15 (93.75%)	0.174
QMG (reduce ≥ 3)	63 (77.78%)	50 (76.92%)	13 (81.25%)	1.000

Score changes are calculated from baseline (Week 0). Response is defined as a reduction of ≥2 points in MG-ADL or ≥3 points in QMG from baseline to Week 4. MG-ADL, Myasthenia Gravis Activities of Daily Living; MGC, Myasthenia Gravis Composite; QMG, Quantitative Myasthenia Gravis; EFG, efgartigimod. *p < 0.05, **p < 0.01.

### 3.3 Multi-cycle treatment patterns

**Figure 2:**
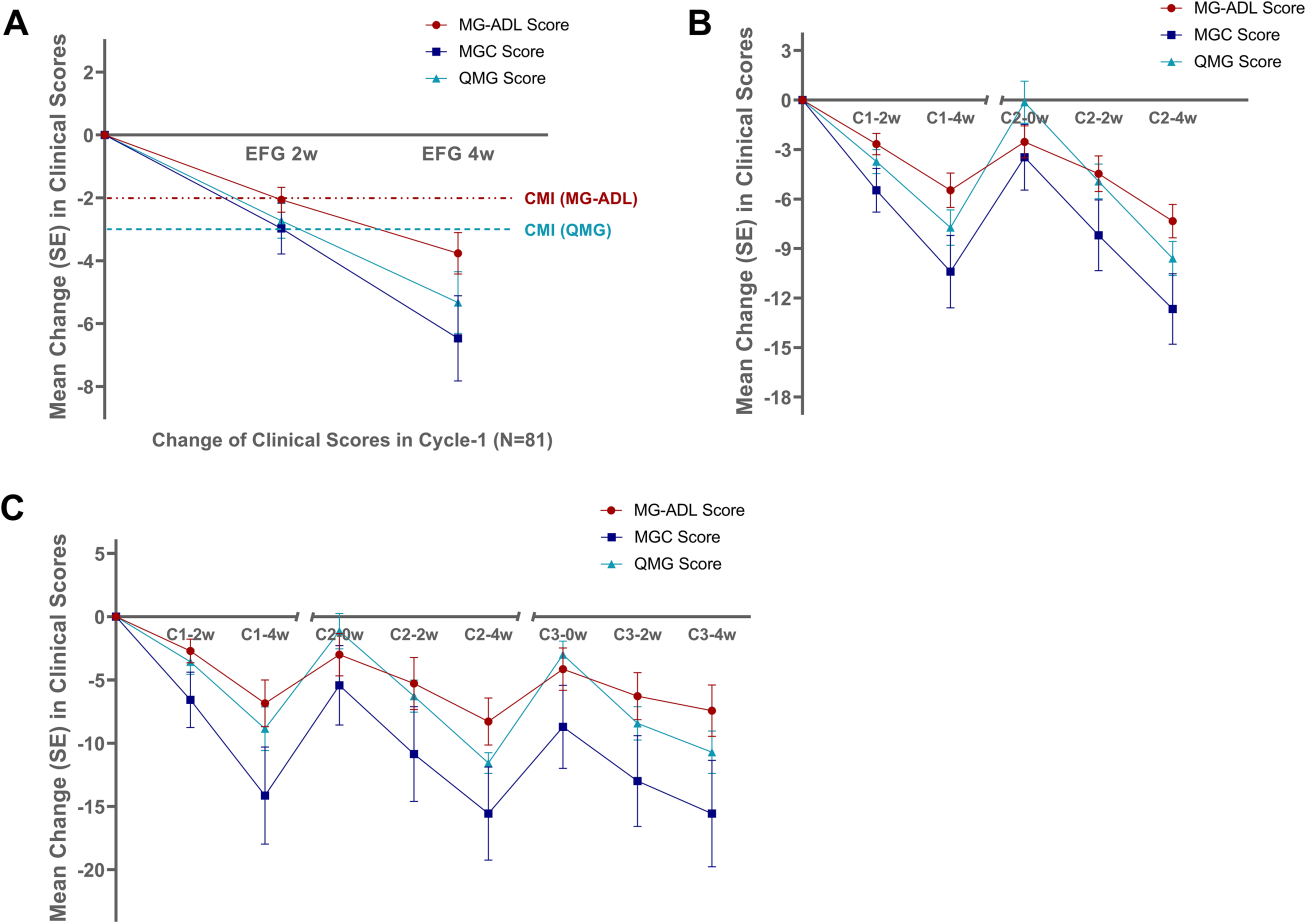
Treatment response in the overall cohort. **(A)** The overall temporal evolution of mean MG-ADL, QMG, and MGC scores from baseline to Week 4 during the first treatment cycle for all enrolled patients (N = 81). Scores were assessed at baseline (Week 0), Week 2, and Week 4. **(B)** Changes of MG-ADL, QMG, and MGC scores for the 16 patients who received two or more treatment cycles, demonstrating from Cycle 1 baseline to Cycle 2. The plot shows the changes at key timepoints: baseline before treatment (0); Cycle 1 Week 2 (C1-2W), Week 4 (C1-4W); Cycle 2 baseline (C2-0W), Week 2 (C2-2W), Week 4 (C2-4W). The y-axis origin (0) represents the pre-treatment baseline at C1-0W. The y-axis origin (0) represents the pre-treatment baseline at C1-0W. **(C)** Changes of MG-ADL, QMG, and MGC scores for the subset of 7 patients who received three or more treatment cycles, showing the progression from Cycle 1 through Cycle 3. Timepoints shown: baseline before treatment (0); Cycle 1 Week 2 (C1-2W), Week 4 (C1-4W); Cycle 2 baseline (C2-0W), Week 2 (C2-2W), Week 4 (C2-4W); Cycle 3 baseline (C3-0W), Week 2 (C3-2W), Week 4 (C3-4W). The y-axis origin (0) represents the pre-treatment baseline at C1-0W. Note: MG-ADL, Myasthenia Gravis Activities of Daily Living; QMG, Quantitative Myasthenia Gravis; MGC, Myasthenia Gravis Composite; W, Week.

In the multi-cycle cohort (N = 16), treatment intervals were determined using the flexible approach detailed in the Methods section. The median interval between the first and second cycles was 54.5 days (range: 29-187). As shown in **Figure 2B**, although clinical recurrence occurred, baseline scores at Cycle 2 initiation were lower than those at Cycle 1 baseline, with the MG-ADL score demonstrating a significant reduction (7.37 ± 2.80 vs. 9.87 ± 4.79, p=0.015). All 16 patients responded favorably to the second cycle, achieving CMI with mean reductions of -5.50 ± 3.89 in MG-ADL and -7.81 ± 4.02 in QMG.

Among these, seven patients received three or more cycles, with a median Cycle 2–3 interval of 52 days (range: 28-75). At Cycle 3 initiation, baseline scores remained lower than Cycle 2 baselines (MG-ADL: 7.14 ± 3.02 vs. 8.28 ± 2.86; MGC: 12.14 ± 6.12 vs. 14.42 ± 5.89; QMG: 16.42 ± 5.18 vs. 18.28 ± 5.24), though these differences were not statistically significant. All seven patients achieved CMI after Cycle 3. One representative patient completed eight cycles and has maintained MM status for four months following efgartigimod discontinuation while on low-dose oral corticosteroids and tacrolimus. No patient in the multi−cycle cohort was lost to follow−up.

### 3.4 Exploration of a reduced-frequency regimen

In our clinical practice, we observed that some elderly patients receiving full-dose efgartigimod, especially alongside other immunosuppressants, experienced a marked IgG reduction with slow recovery, increasing infection risk. Among them, a subset presented with less severe initial symptoms and demonstrated a robust response to efgartigimod. Although they experienced symptom recurrence after the first cycle, their severity remained milder than at baseline. Conventionally, these patients would receive another full cycle, but concerns regarding adverse events and economic burden prompted an exploratory reduced-frequency cycle (RFC, 2 infusions every 2 weeks) in 5 initial patients.

In these 5 patients, this modified regimen effectively maintained disease control without recurrence or significant adverse events. Encouraged by these preliminary results, we summarized the clinical characteristics of these patients to establish candidate selection criteria for further application of RFC. The criteria were as follows: (1) age >65 years; (2) seropositivity for AChR-Ab; (3) baseline MGFA classification of Class III or lower; (4) achievement of Minimal Manifestation status or a clinically significant improvement (reduction ≥4 in MG-ADL and ≥6 in QMG) at the end of the first standard cycle; (5) a pronounced reduction in total IgG during the last standard cycle (LSC), defined as a peak reduction >70% from baseline measured at Week 4 (one week after the fourth infusion); and failure of IgG levels to recover to ≥60% of baseline four weeks after the fourth infusion; (6) concurrent use of a stable dose of another immunosuppressive therapy. Based on these criteria, we subsequently applied RFC to 4 additional patients who had received only one standard cycle of efgartigimod. Thus, a total of 9 patients were included in this exploratory analysis.

The cohort (N = 9) had a median age of 66 years (range: 60-88) with a male-to-female ratio of 2:1. Five patients had received multiple (range: 2-6) standard cycles prior to modification, while four had received only one. All patients had baseline MGFA Class III disease (5 IIIa, 4 IIIb). During the reduced-frequency regimen, all 9 patients remained on a stable dose of concomitant immunosuppressive therapy. Specifically, the mean prednisone dose was 7.5 ± 2.5 mg/day (n=5), mycophenolate mofetil was fixed at 1000 mg/day (n=4), tacrolimus trough levels were maintained within 4–6 ng/mL (n=3), and rituximab was administered 375 mg/m^2^ every 6 months during the RFC period (n=2). Importantly, no patient had an increase in corticosteroid or IST dose during the RFC observation period. Therefore, the sustained efficacy observed is attributable to the modified efgartigimod regimen rather than an escalation of background therapy. All 9 patients in the reduced−frequency subgroup were followed throughout the study period, none was lost to follow−up.

As shown in **Figure 1A**, the baseline MG-ADL score before the first RFC was significantly lower than that before the LSC (7.44 ± 2.65 vs. 10.88 ± 3.65, p<0.001). The median interval between the LSC and the first RFC was 36 days (range: 31-62). After the first RFC, the reduction in MG-ADL was -4.66 ± 1.80, which was less pronounced than that observed after the LSC (-7.33 ± 2.00, p<0.001). However, the absolute MG-ADL score post-RFC (2.77 ± 1.31) was significantly lower than that post-LSC (p=0.021), and 6 of 9 patients achieved MM status. Following the first RFC, 3 patients maintained MM status, while the remaining 6 received a second RFC after a median interval of 42 days (range: 36-58). During the second RFC, the mean MG-ADL reduction was -3.88 ± 1.47, all 6 patients achieved Clinical Minimal Manifestations, and 5 sustained MM status. In these 9 patients, total IgG levels were serially monitored. The percentage change from the baseline of the last standard cycle (LSC−0W) was calculated. After LSC, IgG reached a nadir with a mean reduction of –82.5% ± 7.8%. While four weeks after the fourth infusion (served as the baseline for the first RFC), the mean reduction had partially attenuated to –52.3% ± 9.6%. During the first RFC, the mean reductions were –61.2% ± 7.8% at RFC−2W and –66.6% ± 8.4% at RFC−4W. The parallel changes in IgG and MG−ADL scores are shown in **Figure 1B**.

### 3.5 Safety and treatment disposition

During the treatment and follow-up period, adverse events (AEs) were reported in 65.43% (53/81) of patients. The majority of AEs was mild, with upper respiratory tract infection being the most common (79.24%, 42/53), followed by headache and urinary tract infection. Serious adverse events (SAEs) were observed in only three patients: two patients experienced acute myocardial infarction during infusion, which improved after reperfusion therapy, and one patient died from sudden cardiac death during follow-up after the third treatment cycle. Throughout the study, only five patients required rescue therapy due to suboptimal response. The rescue regimen consisted of intravenous methylprednisolone (1 g/day for 3 days) in three patients, and intravenous immunoglobulin (IVIg, 0.4 g/kg/day for 5 days) in two patients. All five patients belonged to the multi-cycle treatment cohort (≥2 cycles), and rescue therapy was administered between cycles. No patient underwent plasma exchange. No deaths were attributed to myasthenia gravis exacerbation. Clinical improvements were consistently observed not only in AChR-Ab-positive patients but also in those with MuSK-Ab positivity (n=4), double seropositivity for AChR-Ab and MuSK-Ab (n=1), and seronegative status (n=1).

Among the 65 patients who received only one standard treatment cycle, follow-up data on their subsequent clinical management are summarized in **Table 3**. The majority (63.07%, 41/65) maintained adequate disease control with combination immunosuppressive therapy without requiring additional efgartigimod cycles. Among the 41 patients who maintained disease control with combination therapy alone, the maintenance regimen included: low-dose oral corticosteroids (prednisone ≤10 mg/day or equivalent, n=35), mycophenolate mofetil (1000–1500 mg/day, n=9), tacrolimus (2–4 mg/day, n=15), and rituximab (n=6). The doses of these agents remained stable in 36 of 41 patients throughout follow-up, with minor adjustments for tolerability in 5 patients. The median duration of sustained disease control without additional efgartigimod was 7.2 months (range, 3–16 months). A subset (6.15%, 4/65) transitioned to the reduced-frequency regimen described in Section 3.4. Treatment was switched due to inadequate efficacy in 18.46% (12/65) and economic burden in 4.62% (3/65). A small proportion (7.69%, 5/65) were lost to follow-up.

**Table 3:** Post-treatment disposition of patients receiving a single cycle of efgartigimod.

Causes	Number	Percentage
Disease well controlled with combination therapy	41	63.07%
Received reduced-frequency cycle	4	6.15%
Switched treatment due to poor efficacy	12	18.46%
Switched treatment due to economic burden	3	4.62%
Lost to follow-up	5	7.69%
Total	65	100.00%

## 4 Discussion

This single-center, retrospective study provides real-world evidence on the use of efgartigimod in 81 Chinese patients with gMG. Our findings confirm the rapid efficacy of efgartigimod in a real-world setting, with 80.25% of patients achieving an MG-ADL response (reduction ≥2) and 77.78% achieving a QMG response (reduction ≥3) after the first treatment cycle. These response rates are consistent with those reported in the ADAPT trial and other real-world studies across different regions (5–12, 15–18). Notably, clinical improvement was observed not only in AChR-Ab-positive patients but also in those with MuSK-Ab, double seropositivity, and seronegative status, supporting its broad applicability across antibody profiles (19, 20).

A key finding in our cohort was that the majority of patients (80.25%, 65/81) received only a single cycle of efgartigimod. Among these, most (63.07%) maintained adequate disease control with concomitant immunosuppressive therapies—such as corticosteroids, conventional ISTs, or rituximab—without requiring additional efgartigimod cycles. This practice reflects a distinctive treatment strategy adopted at our center: efgartigimod is employed as a short-term induction agent to achieve rapid symptom control, while long-term maintenance is entrusted to conventional immunosuppressants or rituximab. This “induction-maintenance” approach is consistent with previous real-world evidence indicating that efgartigimod provides faster symptom relief than eculizumab and exhibits efficacy comparable to IVIg in patients with impending myasthenic crisis (21–24). Such a strategy may represent a more cost-effective and logistically feasible model, particularly in resource-conscious clinical settings.

Patients who proceeded to multiple treatment cycles (n=16) had significantly higher baseline disease severity. Despite symptomatic recurrence between cycles, the re-initiation baseline scores for subsequent cycles were consistently lower than those of the previous cycle, suggesting a cumulative benefit or a “resetting” of the disease baseline. The individualized, flexible retreatment strategy employed—guided by symptom recurrence and score thresholds rather than a fixed schedule—proved feasible and effective, mirroring the retreatment logic applied in the ADAPT+ study and other real-world cohorts (6, 8).

A notable exploratory finding was the successful implementation of a reduced-frequency regimen (2 infusions per cycle, administered bi-weekly) in a selected subgroup of 9 patients. This modified regimen maintained clinical efficacy, with all patients achieving CMI or MM status, while significantly reducing the infusion burden. The candidate selection criteria—older age, robust initial response, significant IgG reduction, and stable background immunosuppression—may help identify patients suitable for such extended-interval dosing. This exploration addresses a critical practical challenge in long-term FcRn inhibitor use and contributes to the growing discourse on treatment individualization and burden reduction.

The safety profile observed was favorable and consistent with the established profile of efgartigimod (25, 26). Most adverse events were mild and infrequent, with a low incidence of serious adverse events. The fact that only five patients required rescue therapy and none required plasmapheresis further attests to the drug’s effectiveness in preventing severe exacerbations in a real-world population.

This study has several limitations. First, its retrospective, single−center design inherently limits the generalizability of our findings, as the treatment patterns and patient selection reflect the standardized practices of our center. Second, the sample sizes of the multi−cycle and reduced−frequency subgroups were relatively small, which may introduce bias and limit the robustness of subgroup analyses. In particular, the reduced−frequency regimen was explored in a highly selected subgroup of only nine patients; thus, these findings should be considered hypothesis−generating and require validation in larger, prospective cohorts before clinical adoption. Third, the real−world nature of the analysis carries inherent risks of selection bias and the absence of a standardized protocol for retreatment or regimen modification. Longer follow−up and prospective, controlled studies are needed to better define the optimal sequencing and combination of efgartigimod with other immunosuppressive agents, and to validate the reduced−frequency regimen.

Contextualized within the global literature, our experience reinforces the efficacy of efgartigimod across diverse patient populations. It also highlights a potential divergence in treatment paradigms. While many international cohorts describe a model of repeated cycles as chronic maintenance therapy, our data illustrate that a significant proportion of patients can be effectively managed with a single cycle as part of a combined immunomodulatory strategy. The successful exploration of a reduced-frequency regimen also offers a promising approach to minimizing treatment burden. This observation invites further investigation into predictive biomarkers for identifying patients who will require multiple cycles versus those who can be sustained on conventional ISTs after initial rapid control. Moreover, while our study was not designed for direct head−to−head comparison, the rapid onset of action observed (mean MG−ADL reduction of -2.06 by Week 2) appears comparable to that reported for IVIg in impending myasthenic crisis (21) and for eculizumab in refractory gMG (27). Prospective comparative studies are needed to determine the relative speed of response among efgartigimod, IVIg and plasma exchange.

## Data Availability

The original contributions presented in the study are included in the article/supplementary material. Further inquiries can be directed to the corresponding authors.
